# Spontaneous Slow Fluctuation of EEG Alpha Rhythm Reflects Activity in Deep-Brain Structures: A Simultaneous EEG-fMRI Study

**DOI:** 10.1371/journal.pone.0066869

**Published:** 2013-06-18

**Authors:** Kei Omata, Takashi Hanakawa, Masako Morimoto, Manabu Honda

**Affiliations:** 1 Department of Functional Brain Research, National Institute of Neuroscience, National Center of Neurology and Psychiatry, Kodaira, Tokyo, Japan; 2 Department of Biofunctional Imaging, Medical Photonics Research Center, Hamamatsu University School of Medicine, Hamamatsu, Shizuoka, Japan; 3 Integrative Brain Imaging Center, National Center of Neurology and Psychiatry, Kodaira, Tokyo, Japan; 4 Japan Science and Technology Agency, PRESTO, Saitama, Japan; 5 Japan Science and Technology Agency, CREST, Saitama, Japan; Hangzhou Normal University, China

## Abstract

The emergence of the occipital alpha rhythm on brain electroencephalogram (EEG) is associated with brain activity in the cerebral neocortex and deep brain structures. To further understand the mechanisms of alpha rhythm power fluctuation, we performed simultaneous EEGs and functional magnetic resonance imaging recordings in human subjects during a resting state and explored the dynamic relationship between alpha power fluctuation and blood oxygenation level-dependent (BOLD) signals of the brain. Based on the frequency characteristics of the alpha power time series (APTS) during 20-minute EEG recordings, we divided the APTS into two components: fast fluctuation (0.04–0.167 Hz) and slow fluctuation (0–0.04 Hz). Analysis of the correlation between the MRI signal and each component revealed that the slow fluctuation component of alpha power was positively correlated with BOLD signal changes in the brain stem and the medial part of the thalamus and anterior cingulate cortex, while the fast fluctuation component was correlated with the lateral part of the thalamus and the anterior cingulate cortex, but not the brain stem. In summary, these data suggest that different subcortical structures contribute to slow and fast modulations of alpha spectra on brain EEG.

## Introduction

Spontaneous electroencephalogram (EEG) is widely used as a clinical tool to judge the general condition of the brain, such as the stage of sleep or level of consciousness. The EEG rhythm that ranges from 8 to 13 Hz when recorded from the occipital area during a resting state with the eyes closed is termed the alpha rhythm [1] or posterior dominant rhythm. The alpha rhythm is generally considered an index of vigilance or arousal, and the emergence of alpha oscillations is thought to represent an “idling” state of the relevant cortices [2], [3]. In addition, the alpha rhythm is now widely used as an index of evaluation for relaxation or pleasure in various fields such as neuromarketing [4]–[6].

Previous studies using multimodal methods, especially simultaneous EEG recordings and neuroimaging procedures, have attempted to identify the areas of the brain correlated with the power of the alpha rhythm [7]–[17]. In general, negative correlations between alpha power and brain activity have been reported within the cerebral neocortex, especially the occipital, parietal, and inferior frontal regions, whereas positive correlations have been observed within the central deep-lying brain regions such as the thalamus, amygdala, and insula as well as the anterior cingulate cortex and cerebellum.

The negative correlation between cortical activation and the EEG in the alpha frequency range is a relatively common finding across previous studies. It is well established that the power of the alpha rhythm decreases when cortical activity beneath the EEG electrode increases, including alpha attenuation [1] and event-related desynchronization (ERD) [18]. Recently, this relationship was applied to the field of brain–machine/computer interface (e.g. [19]). Conversely, positive correlations between the alpha rhythm and brain activity by fMRI are not always reported and the causes remain unclear, which may be partly due to inaccuracy in the assumption of a fixed canonical HRF as shown by De Munck et al. [20], [21].

The spontaneous fluctuation of alpha power is likely to reflect a mixture of multiple factors, each having a different dynamic characteristic. First, the generation and modulation of alpha rhythm is thought to involve different brain regions. Salek-Haddadi et al. [22] reported that “alpha oscillations may be related to three different types of areas: (1) the generators of the cortical rhythm, such as the occipital cortex; (2) areas forming part of the circuit but not directly generating the scalp-detectable rhythms (e.g. thalamus); and (3) other areas correlated with alpha but not causally linked, for example as linked to changes in arousal only.” Second, the transition of alpha oscillation has some different dynamics. For example, a phenomenon known as “waxing and waning” of the alpha rhythm occurs for a period of several seconds [23]. Moreover, the ERD occurs within seconds after stimuli [3]. Furthermore, the arousal level characterized by alpha oscillation [24] is altered very slowly and has a longer time constant.

Thus, if different brain systems regulate alpha rhythm in parallel, the alpha power time series (APTS) on EEG may consist of different dynamic components of alpha power. To test this hypothesis, we performed simultaneous EEG and fMRI to record the alpha oscillation and brain activity during a resting state. By applying a data-driven method known as empirical mode decomposition (EMD) [25] and low and high pass filters to EEG data to separate the APTS into several components, we examined the relationship between the different frequency components of the alpha power time series (APTS) on EEG and brain activity to determine the dynamics of the relevant brain regions in alpha power fluctuation.

In the present study, we focused on the positive correlation between the alpha rhythm and brain activity for practical use of EEG signals to monitor activity in deep-lying brain regions. These regions of the brain are known to be involved in diffuse regulation by means of widely modulating neuronal responses through diffuse projections from the brain stem to various parts of the brain, such as the reticular formation [26]. By determining the relationship between EEG signals and deep-lying brain region activity, scalp EEG may be used as a practical index of activity of deep brain structures without functional magnetic resonance imaging (fMRI).

## Materials and Methods

### Subjects

Twenty healthy volunteers participated in this study (9 female and 11 male subjects; mean age, 27.3 years). The subjects gave written informed consent before the experiments, which were approved by the institutional ethical review board of the National Institute of Neuroscience, National Center of Neurology and Psychiatry. According to the approved protocol, subjects with a current or previous history of neurological or psychiatric disorders and those with metal implantation were excluded from the study. The subjects were asked to lie still on a scanner bed in the dark for 20 minutes with their eyes closed, but not fall asleep, to obtain spontaneous variations in the alpha rhythm.

### Measurements of simultaneous EEG and fMRI

EEGs were recorded with a 32-channel MR-compatible EEG amplifier (Brain Products, Munich, Germany) and an EEG cap with Ag/AgCl electrodes according to international standards (10/20 system). To correct ballistocardiogram artifacts, electrocardiographic data were also captured from electrodes on the backs of subjects. The reference electrode for the EEG recording was positioned between Fz and Cz. EEG data were acquired at a rate of 5 KHz using BrainVision Recorder software (Brain Products). The EEG amplifier had an amplitude resolution of 16 bits. A vacuum cushion was used to fix the subject's head within the head coil to avoid artifacts originating from subject movement and the ballistocardiogram [c.f.] [27]. The amplifier system was placed beside the subject's head within the scanner during fMRI to shorten the cable between the EEG cap and the amplifier.

MRI was performed with a 3-Tesla scanner (Trio; Siemens, Erlangen, Germany) using a standard transmitter-receiver coil. The T1-weighted sequence, MPRAGE, was used for anatomic referencing of the fMRI recordings and co-registration (TR, 2000 ms; TE, 4.4 ms; FA, 80 degrees; voxel size, 1×1×1 mm; 196 slices). For functional scans, T2*-weighted, gradient-echo, echo planar imaging was used (TR, 3000 ms; TE, 30 ms; FA, 90 degrees; voxel size, 3×3×3 mm; 40 slices). A total of 404 image volumes were acquired at the rate of one every 3 seconds. The first four volumes were discarded to avoid magnetic saturation effects. The total time per session was 20 minutes.

To establish time alignment between the EEG data and blood oxygenation level-dependent (BOLD) signals, a SyncBox device (Brain Products) was used to achieve phase synchrony between the clock for digital sampling of the EEG data and that for driving the MR systems gradient switching. Thus, the starting point of MR image acquisition in each interval was marked in the EEG time course data in which data sampling points were precisely synchronized with MR image acquisition. These markers were used for MRI scanner artifact correction, as described below.

### Analysis of EEG data

To correct artifacts originating from the MRI scanner and ballistocardiogram, the recorded EEG data were processed by BrainVision Analyzer 2.0 (Brain Products) using the average template subtraction method [28], [29]. First, all data were filtered by a low-pass filter with a cut-off frequency of 70 Hz. Because MRI scanner artifacts were regularly repeated every TR interval, the interval of these artifacts could be precisely identified from the markers of the starting point of each MR image acquisition. A template of MRI scanner artifacts in EEG signals was created by averaging the MRI scanner artifacts over a set number of intervals and subsequently subtracting this average from the data. The sampling rate of the data was decreased to 250 Hz. Second, the ballistocardiogram artifacts were removed in a similar fashion. The peaks of the R-waves detected in the electrocardiographic channel were marked by a cross-correlation between a semi-automatically defined pulse peak and the data. A template was created by averaging the EEG data time-locked to the timing of the detected R-wave peaks, and then the averaged template was subtracted from the original EEG data for each R-wave peak. We also employed an independent component analysis (ICA) [30], [31] and obtained a similar result. This compared well with a report by Grouiller et al. [32] showing that the template subtraction method is efficient in removing artifacts for experimental data.

**Figure 1:**
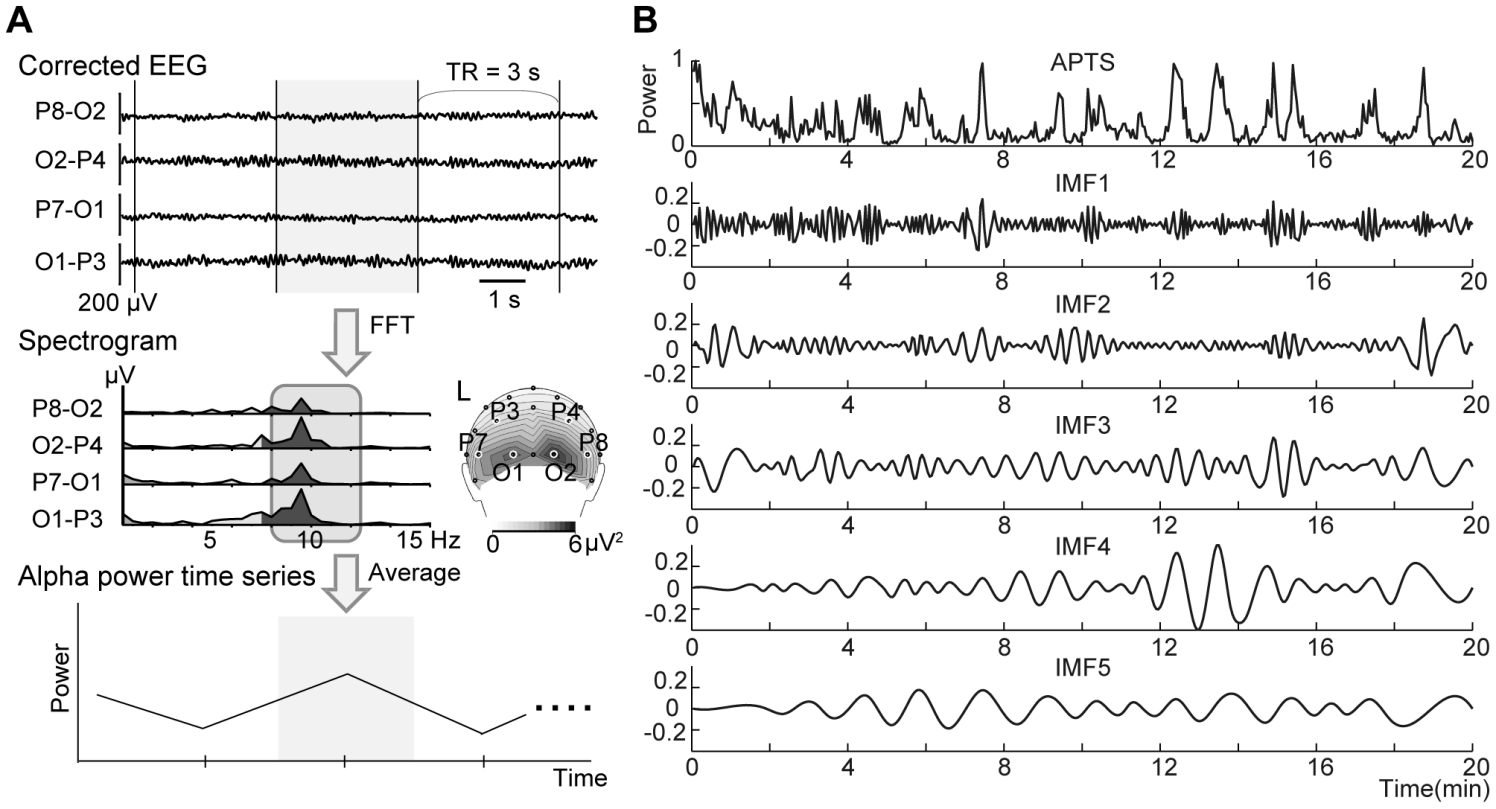
Calculation of the EEG alpha power time series (APTS) and intrinsic mode functions (IMFs). A: After removal of the MRI and ballistocardiogram artifacts, the EEG data from the four bipolar channels were subjected to frequency analysis using fast Fourier transform (FFT) for each 3-second segment (gray in the upper panel). The powers of the alpha band across the four bipolar channels were averaged. The averaged power values were then temporally aligned as the APTS, as shown in the bottom panel. A scalp topography of alpha power of a single subject is shown in the right middle panel. Note that the topography is described by EEG data of a unipolar induction, and L indicates the left side of the brain. B: An example of the IMFs for a single subject. An APTS of a single subject is shown in the upper panel. Next, the IMFs separated by the empirical mode decomposition (EMD) from the APTS were shown from the first to the fifth IMF.

To avoid bias effects from the reference positions (e.g., TP9, TP10, FCz), and to specify the alpha power of the parieto-occipital regions, the corrected EEG data recorded from the parieto-occipital regions (i.e., P3, P4, P7, P8, O1, and O2 in the international 10/20 system) were reconfigured into four bipolar derivations (i.e., P7–O1, O1–P3, P8–O2, and O2–P4) after correction of the MRI scanner and ballistocardiogram artifacts. The re-reference to the parieto-occipital regions emphasizes the relevant local EEG sources over global EEG sources by removing signals that are common between the neighboring electrodes. The data were segmented every 3 seconds to match the TR of the fMRI data. The powers of the frequency components in these four channels were calculated by fast Fourier transformation (FFT) with a frequency resolution of 0.5 Hz. A moving time window of 2-second lengths with interpolation was used to calculate the FFT of 3-second analysis epochs to ensure the alpha frequency range from 8 to 12.5 Hz. The powers of the alpha rhythm band (8–12.5 Hz) for each channel were averaged for each segment. Averaging the powers across hemispheres emphasizes or assumes commonality in this measure across hemispheres. This procedure resulted in an average alpha band power every 3 seconds, denoted as the APTS (Figure 1A). The data points in the APTS exceeding the standard deviation by 3-fold or greater were excluded and replaced by linearly interpolated values. To exclude systematic differences in the amplitude of the APTS across subjects, the APTS in each subject was normalized to the range of 0 to 1, as follows [33]:
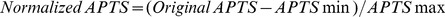



Here, APTSmin and APTSmax represent the minimum and maximum values of APTS, respectively. The original APTS was used as a regressor in the general linear model (GLM) for fMRI analysis to explore the brain regions whose activity specifically correlated with the original APTS. We assumed that the APTS may reflect a different type of brain mechanism of generation or modulation of the alpha EEG. To examine for such a mechanism, we focused on the dynamic aspect of the APTS changes and analyzed the frequency components of the APTS. To divide the APTS into sub-frequency components, a data-driven method termed empirical mode decomposition (EMD) introduced by Huang et al. [25] was employed. The EMD is an algorithm whereby a single time-course is decomposed into its oscillatory components and is applied to non-stationary and nonlinear time series analysis [25], such as APTS and BOLD signals. For example, Niazy et al. [34] reported the ability of the EMD for investigating time series of spontaneous BOLD signals during resting-state. Each oscillatory component is called an intrinsic mode function (IMF) that is defined by the following two conditions. First, the number of zero-crossings and extrema must be the same or differ at most by 1. Next, the mean between the upper and lower envelopes must be close to zero according as stopping criteria.

The algorithm of EMD [35] can be described as follows:

Given a signal *x(t),*

1. Identify all extrema of *x(t)*
2. Interpolate between minima (resp. maxima), resulting in an envelope *e_min_(t)* (resp. *e_max_(t)*)
3. Compute the mean *m(t)* = (*e_min_(t)*+*e_max_(t)*)/2
4. Extract the detail *d(t)* = *x(t)*−*m(t)*
5. Iterate on the residual *m(t)*

Steps 1 to 4 are iterated until the detail satisfies the above two conditions. This procedure is defined as a sifting process [25], [35], [36]. The detail is referred to as an IMF after the sifting process stops, the residual is calculated, and step 5 is followed.

**Figure 2:**
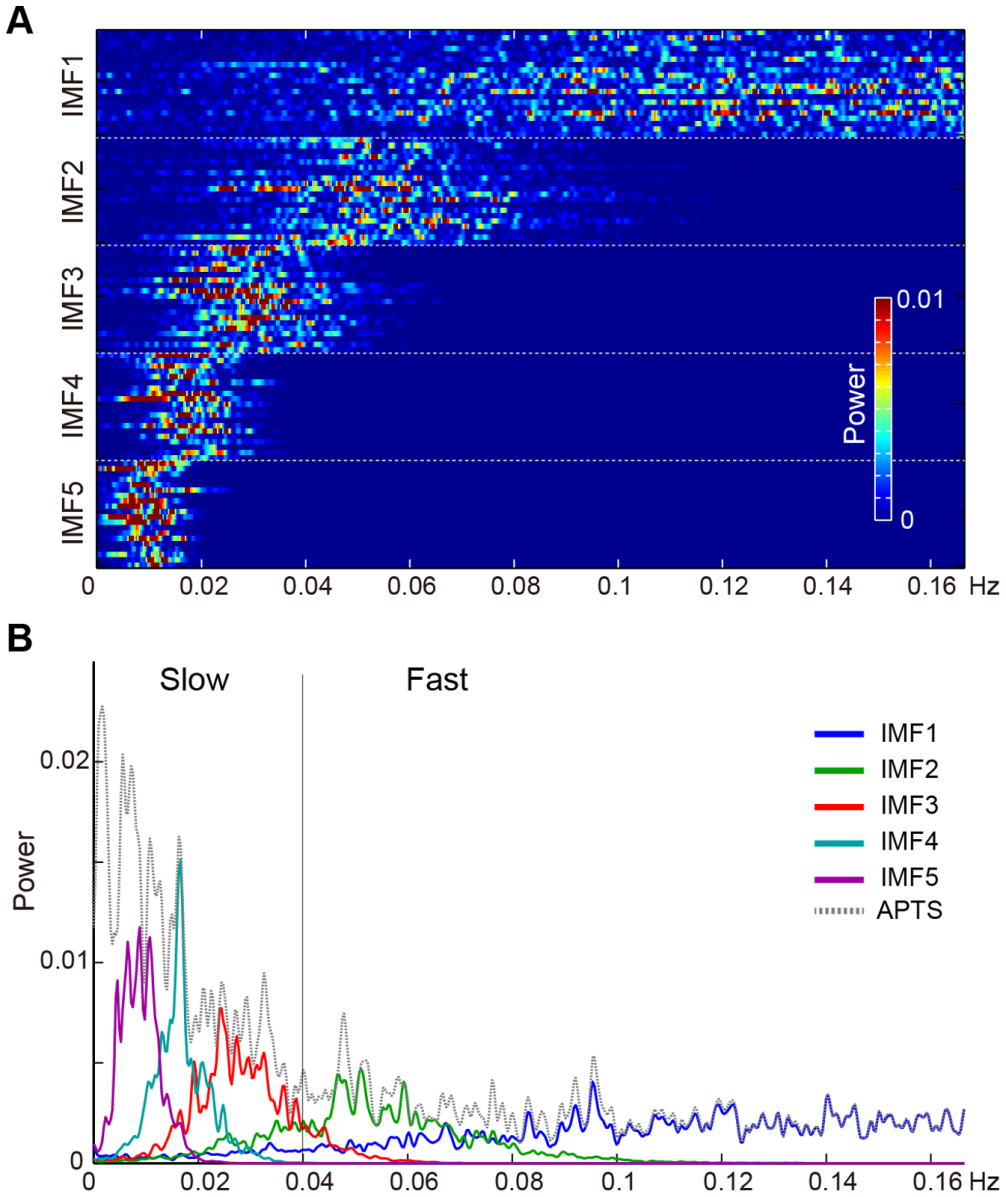
Averaged power spectrums of the IMFs during 20 minutes of fMRI scanning. A: Distribution of the frequency of all IMFs for each subject. Color illustrates the power of the IMFs from 0–0.01. Each line within IMFs represents the frequency spectrum of each subject (total of 20 subjects). B: The averaged power spectrum of the APTS and the IMFs across all subjects. The dashed line represents the averaged power spectrum of the detrended APTS across all subjects. The colors of the profiles represent the spectrum of each IMF as follows. IMF1: blue, IMF2: green, IMF3: red, IMF4: cyan, IMF5: violet. Slow and Fast indicate the frequency ranges of the slow and fast fluctuation components, respectively. 0.04 Hz was the border of the segmentation.

The APTS was subjected to the EMD algorithm to explore its IMFs. Figure 1B depicts the application of the EMD to the APTS of a single subject. In the present study, we chose IMFs 1 to 5 for further analyses. The IMFs were subjected to FFT analysis to explore the frequency profile. The Nyquist frequency of the FFT was 0.167 Hz as the APTS was sampled every 3 seconds. Figure 2A depicts all the spectrums of the IMFs of each subject. Each IMF group derived from different subjects roughly covered the same frequency band (Figure 2A). Figure 2B shows the averaged power spectrums of each IMF across all subjects. It is worth noting that each IMF has a unique frequency band (the first IMF covers the highest frequencies and the last IMF covers the lowest one), and that each frequency band of the IMFs have crossovers with each other. These IMFs were used as regressors in the GLM for fMRI analysis to explore the brain regions whose activity specifically correlated with each IMF.

According to the results of the correlation between the IMFs and brain activity, to verify the results we divided the APTS into slow and fast fluctuation components of the APTS using low and high pass filters. Based on the crossover between the averaged power spectrum of IMF2 and IMF3, the two fluctuation components of the APTS were defined as follows: slow fluctuation(<0.04 Hz) and fast fluctuation (>0.04 Hz) (Figure 2B). Each fluctuation component was then extracted by filtering the original APTS with Butterworth low-pass and high-pass filters (low-pass filter: passband ripple, 3 dB; passband frequency, 0.04 Hz; slope, −49 dB per octave; high-pass filter: passband ripple, 3 dB; passband frequency, 0.04 Hz; slope, 48 dB per octave). These two fluctuation components were used as regressors in the GLM for fMRI analysis to explore the brain regions whose activity specifically correlated with each fluctuation component, as described below. Note that the slow and fast APTS components and the slow (8–10 Hz) and fast (10–13 Hz) alpha rhythms should be not be confused with each other. The fast alpha rhythm refers to the frequency components of the raw EEG waveform, while the slow alpha rhythm refers to the frequency components in the longer trend of the power of the alpha frequency band of the EEG.

### Analysis of fMRI data

fMRI data were analyzed with SPM5 on MATLAB (MathWorks, Natick, MA, USA). Preprocessing of the fMRI included slice timing correction, realignment, spatial normalization, and spatial smoothing with an 8-mm, three-dimensional Gaussian filter [37]. The brain regions whose BOLD signals were correlated with the EEG components, namely the APTS and each of its components, were statistically evaluated with a general linear model [37] in which both the explanatory variables of interest and those of non-interest were used as multiple regressors. Each original APTS, its IMFs, and its slow and fast fluctuation components were convolved with the canonical hemodynamic response function to take into account hemodynamic delay and dispersion of BOLD signals, and then used as an explanatory variable. The six realignment parameters were used as variables of non-interest to remove the effect of head motion on MRI signals. The MRI signal of cerebrospinal fluid (CSF) was also used as an explanatory variable of non-interest to exclude signals originating from vessels and ventricular areas reflecting cardiac beats and respiration [38]–[40] that were irrelevant to the neural activities. The CSF signal was calculated by averaging the MRI signal in the ventricles, which were anatomically defined by the segmentation function of SPM5. When taken together, we conducted three types of GLM, with (i) the GLM of the original APTS, (ii) its IMFs, and (iii) its slow and fast fluctuation of the APTS, estimated separately. Each GLM included the six realignment parameters and the CSF signal as nuisance covariates.

For each GLM, at the first level the contrast images corresponding to the regressors were created for each subject and entered into a second level one-sample t test. Additionally, for the third GLM, a paired t test was conducted to determine whether the contrast weights were significantly different between the slow and fast fluctuation components within regions of interest inclusively masked by brain regions that positively correlated with either the slow or fast fluctuation components. For all data, a threshold of uncorrected p<0.001 for peak-level and a cluster-level family-wise error (FWE) of 0.05 [41] were used for statistical analyses. An atlas of the human brain was used as an anatomical reference for the deep-lying brain regions [42].

## Results

**Figure 3:**
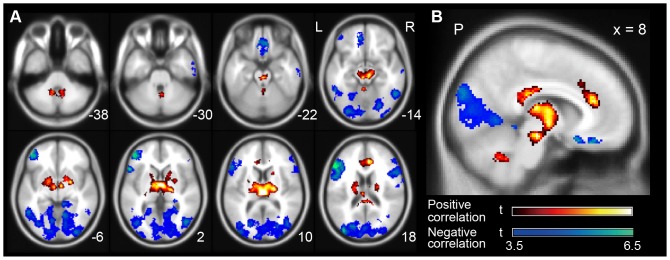
Group analysis of the correlations between alpha power fluctuation and the BOLD signal on fMRI. *A*: The positive (red-white) and negative (blue-green) correlation maps in the multiple axial planes are superimposed on a standard brain template according to the Montreal Neurological Institute (MNI) coordinate [61]. The number in the bottom right of each slice indicates a Z coordinate in the MNI space. *B*: The positive and negative correlation maps in the sagittal planes at an X coordinate of +8 mm in the MNI coordinate. Only the areas with a peak-level uncorrected p<0.001 and a cluster-level FWE of 0.05 by random-effect analysis are shown. The color bars show t-values between 3.5 and 6.5. The letters in the figure indicate the direction of each brain image (L: left; R: right; P: posterior).

In this manuscript, we use the term “correlation” to explain the relationships between the explainable values and the brain activity in the GLM. The original APTS was positively correlated with brain activity in the thalamus, anterior cingulate cortex, brain stem, and cerebellum and was negatively correlated with activity in the broad areas of the cerebral cortex (the superior parietal lobule, cuneus, middle occipital gyrus, middle frontal gyrus, rectal gyrus, and inferior temporal gyrus) (uncorrected p<0.001, extent >103 voxels) (Figure 3, Table 1). These findings are generally concordant with those of previous reports [7], [11], [12], [15]–[17].

**Table 1:** Brain regions whose activity correlated with the power of the EEG alpha rhythm (*p*-value, cluster-level FWE of 0.05).

Correlation	Brain region	Side	Local maximum point					
			t-value	X	Y	Z	P value	Clustersize
positive	brainstem	–	9.65	4	−26	−18	<0.001	4136
	thalamus	bilateral	7.47	−2	−22	10	<0.001	
			7.07	2	−6	2	<0.001	
	anterior cingulate cortex	bilateral	6.36	4	34	22	<0.001	686
	cerebellum	left	5.65	−10	−54	−40	<0.001	395
	cerebellar vermis	right	5.34	4	−56	−32	<0.001	
negative	superior parietal lobule, cuneus, middle occipital gyrus	bilateral	7.94	34	−50	56	<0.001	28926
	middle frontal gyrus	left	7.32	−44	46	0	<0.001	
	rectal gyrus	bilateral	6.46	−12	44	−16	<0.001	608
	middle frontal gyrus	right	5.68	24	30	44	0.001	211
	Inferior temporal gyrus	bilateral	5.52	56	−54	−12	<0.001	299
	Inferior temporal gyrus	right	5.19	62	−12	−26	<0.001	162

**Figure 4:**
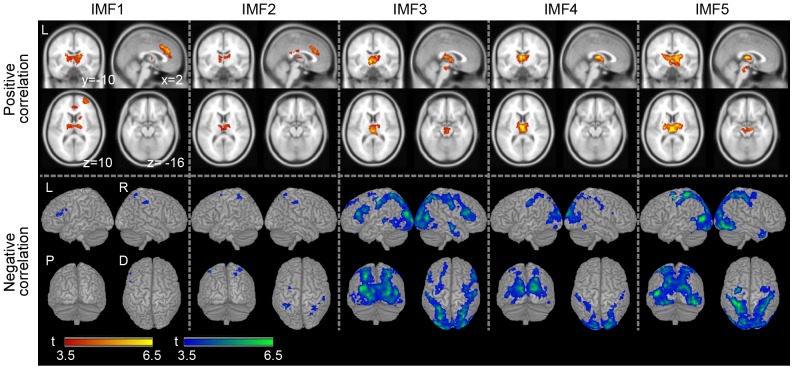
Group analysis of the correlations between IMFs and the BOLD signal on fMRI. In the upper panel, the positive (red-yellow) correlation maps in the multiple axial planes are superimposed on a standard brain template according to the Montreal Neurological Institute (MNI) coordinate [61]. The positive correlation maps for each IMF are shown in the sagittal planes at an X coordinate of +2 mm, a Y coordinate of −10 mm, and a Z coordinate of 10 mm and −16 mm in the MNI coordinate. In the bottom panel, the negative (blue-green) correlation maps for each IMF are rendered on a standard template brain image. Only the areas with a peak-level uncorrected p<0.001 and a cluster-level FWE of 0.05 by random-effect analysis are shown. The color bars show t-values between 3.5 and 6.5. The letters in the figure indicate the direction of each brain image (L: left; R: right; P: posterior; D: dorsal).

Figure 4 illustrates the brain regions with activities that correlated with the IMFs. Tables 2 and 3 show the details of the positive and negative correlated areas, respectively. While the threshold of uncorrected p<0.001 for peak-level was used, the extent threshold that was equal to a FWE of 0.05 was different from each IMF (IMF1: extent >96; IMF2: extent >130; IMF3: extent >114; IMF4: extent >166; IMF5: extent >128). The results of the positive correlation with the IMFs are shown in the upper part of Figure 4. The IMF1 was correlated with activity in the anterior-lateral part of the thalamus, the anterior cingulate cortex, the dorsolateral prefrontal cortex, the cerebellum, and the caudate nucleus. Similarly, the IMF2 was correlated with activity in the anterior cingulate cortex and the anterior part of the thalamus. Conversely, the IMF3 was correlated with activity in the medial part of the thalamus and the brain stem. The IMF4 was correlated with activity in the medial dorsal part of the thalamus. Furthermore, the IMF5 was correlated with activity in the lateral and medial part of the thalamus and brain stem (Figure 4 and Table 2). In summary, both IMF1 and 2, including the higher frequency band of the APTS, were positively correlated with brain activity in the anterior cingulate cortex and the anterior-lateral part of the thalamus, whereas the IMF3, 4 and 5, including the lower frequency band of the APTS, were positively correlated with brain activity in the medial-dorsal part of the thalamus and/or the brain stem.

**Table 2:** Brain regions whose activity positively correlated with the IMFs components of the EEG alpha power (*p*-value, cluster-level FWE of 0.05).

IMF components	Brain region	Side	Local maximum point					
			t-value	X	Y	Z	P-value	Cluster size
IMF1	thalamus	bilateral	6.31	20	−14	14	<0.001	1159
	anterior cingulate cortex	bilateral	6.24	4	34	26	<0.001	1007
	dorsolateral prefrontal cortex	right	6.07	28	60	14	<0.001	519
	cerebellum	left	5.69	−36	−58	−38	0.001	202
	caudate nucleus	right	4.68	20	12	14	0.032	106
IMF2	anterior cingulate cortex	bilateral	5.47	2	18	36	<0.001	543
	thalamus	left	4.90	−8	−10	0	<0.001	382
		right	4.75	6	−12	10		
IMF3	thalamus	left	6.47	−6	−10	0	<0.001	1332
		right	6.14	4	−12	0		
	brain stem	–	4.82	2	−24	−16		
IMF4	thalamus	bilateral	7.46	−4	−12	12	<0.001	1248
		left	7.32	−4	−4	4		
IMF5	thalamus	bilateral	7.20	0	−20	10	<0.001	2513
		left	6.68	−22	−16	10		
	brain stem	–	5.43	4	−28	−26		
	cerebellum	left	5.66	−28	−70	−32	0.003	225
	supramarginal gyrus	right	5.00	56	−38	42	0.014	171

**Table 3:** Brain regions whose activity negatively correlated with the IMFs components of the EEG alpha power (*p*-value, cluster-level FWE of 0.05).

IMF components	Brain region	Side	Local maximum point					
			t-value	X	Y	Z	P-value	Cluster size
IMF1	inferior frontal cortex	left	4.73	−52	24	18	0.011	132
IMF2	superior parietal lobe	left	6.07	−38	−48	58	<0.001	320
		right	5.05	48	−30	42	<0.001	555
	precentral gyrus	right	5.57	30	−2	48	0.032	145
		left	4.78	−28	−6	52	0.004	222
IMF3	occipitoparietal cortex	right	10.35	30	−60	32	<0.001	31596
	inferior frontal cortex	left	7.14	−46	4	30	<0.001	1937
	orbitofrontal cortex	left	6.70	−12	50	−10	<0.001	441
	middle temporal gyrus	right	6.11	64	−12	−16	<0.001	300
IMF4	occipito-parietal cortex	right	7.73	22	−86	18	<0.001	11545
	inferior temporal gyrus	right	6.30	52	−50	−10	0.006	266
	inferior frontal gyrus	right	5.16	46	10	22	0.036	180
IMF5	middle occipital gyrus	left	9.43	−44	−76	6	<0.001	17145
	precentral gyrus	right	7.73	−40	−14	58	<0.001	433
	medial temporal pole	right	5.62	46	12	−40	0.002	253
	middle orbital gyrus	bilateral	5.14	−4	54	−10	0.041	134

The results of the negative correlation with the IMFs are shown in the bottom part of Figure 4. The negative correlation with each IMF component was found within the occipitoparietal cortex, but not in all the IMF components. Although the results of the IMF1 and IMF2 showed a small amount of negative correlation in the brain regions, IMF3, 4, and 5 were widely negatively correlated with activity in the occipital-parietal cortex. The IMF1 was correlated with activity in the left inferior frontal cortex, and the IMF2 was correlated with activity in the superior parietal lobe and precentral gyrus. Subsequently, the IMF3 was correlated with activity in the occipitoparietal cortex, the inferior frontal cortex, the orbitofrontal cortex, and the middle temporal gyrus. The IMF4 was correlated with activity in the occipitoparietal cortex, the inferior temporal gyrus, and the inferior frontal gyrus. Furthermore, the IMF5 was correlated with activity in the middle occipital gyrus, the precentral gyrus, the medial temporal pole, and the middle orbital gyrus (Figure 4 and Table 3).

**Figure 5:**
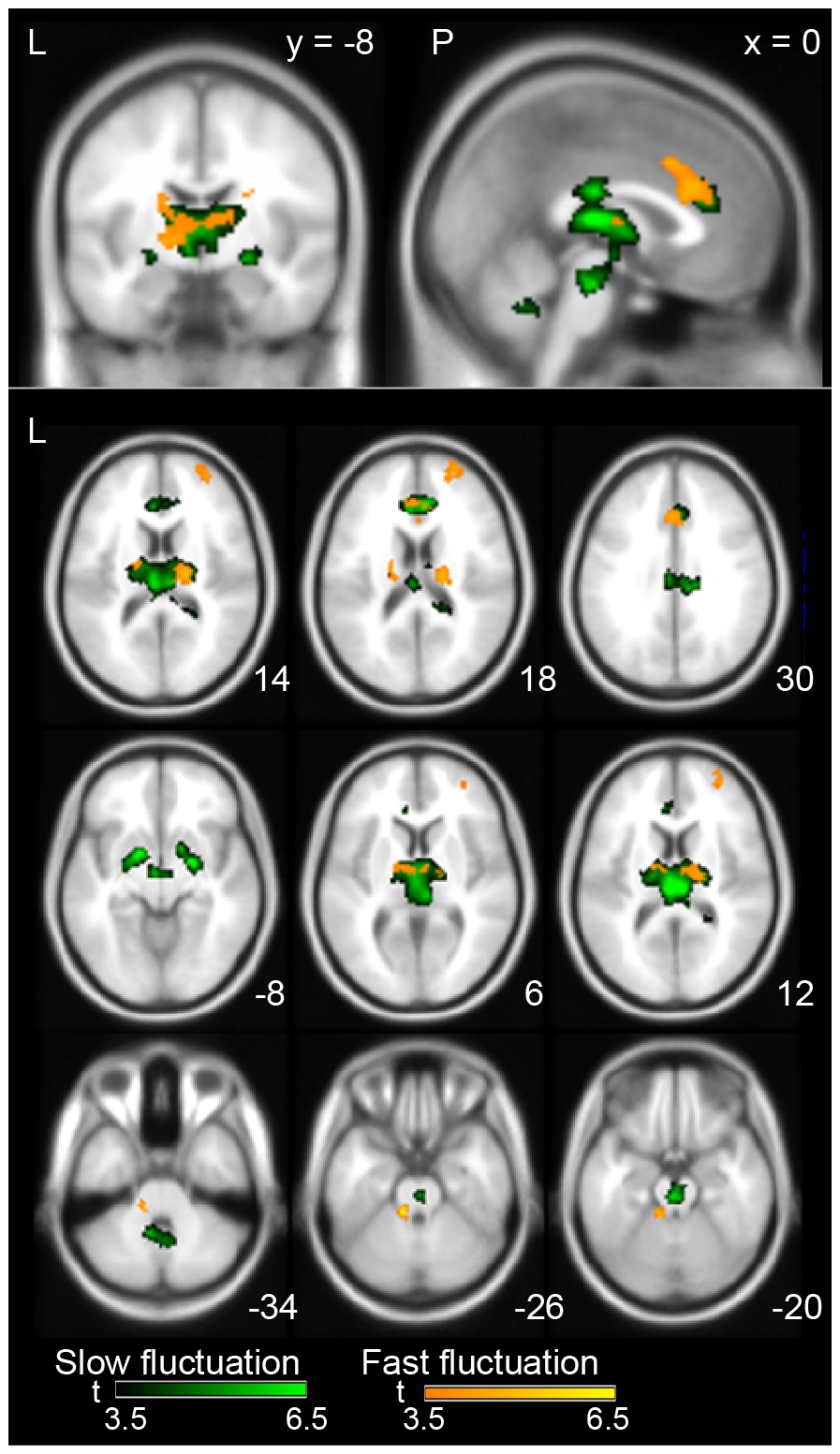
Positive correlation maps between the slow and fast fluctuation of the APTS and the BOLD signal. Only the areas with a peak-level uncorrected p<0.001 and a cluster-level FWE of 0.05 are shown in the random-effect analysis. Statistical results are superimposed on an averaged MRI. The green and orange colors on the brain images indicate the correlation between the BOLD signals and the slow and fast fluctuation components, respectively. The color bars at the bottom of the figure show t-values between 3.5 and 6.5. Numbers in the bottom right of each slice show the coordinates according to the MNI space. Upper: Sagittal and coronal planes. Lower: Multiple axial planes. The letters in the figure indicate the direction of each brain image (L: left; P: posterior).

More importantly, the slow and fast fluctuation components of the APTS showed a specific relationship with brain activity (uncorrected, p<0.001 and extent >131 voxels for the slow fluctuation and 75 voxels for the fast fluctuation, respectively). Figure 5 illustrates the brain regions with activities that correlated with either slow or fast fluctuation. Table 4 gives details of the correlated areas. Slow fluctuation was correlated with activity in the medial part of the thalamus and brain stem, the anterior cingulate cortex, the amygdalae, and the cerebellum. By contrast, the fast fluctuation component was correlated with activity in the cerebellum, the anterior and middle cingulate cortex, the superior frontal cortex, and the lateral part of the thalamus.

**Table 4:** Brain regions whose activity correlated with the slow and fast fluctuation components of the EEG alpha power and the comparison between the slow and fast fluctuation components (*p*-value, cluster-level FWE of 0.05).

Fluctuation component	Brain region	Side	Local maximum point					
			t-value	X	Y	Z	P-value	Cluster size
slow	thalamus	bilateral	7.58	6	−24	10	<0.001	2861
	brainstem	bilateral	5.69	0	−22	−22		
	anterior cingulate cortex	bilateral	7.29	6	32	20	<0.001	658
	amygdala	right	7.18	24	−4	−8	0.004	258
		left	7.09	−16	0	−8	0.004	261
	cerebellum	bilateral	5.38	−10	−54	−36	0.006	241
fast	cerebellum	left	7.51	−10	−38	−26	0.024	134
	anterior and middle cingulate cortex	bilateral	5.95	6	18	38	<0.001	489
	superior frontal cortex	right	5.37	28	54	24	0.002	222
	thalamus	right	5.07	18	−16	16	<0.001	487
		left	4.67	−12	−8	0		
slow > fast	thalamus	bilateral	7.97	4	−26	8	<0.001	848
			5.35	−4	−10	−8		
	brainstem	bilateral	5.79	2	−20	−22	0.045	139

**Figure 6:**
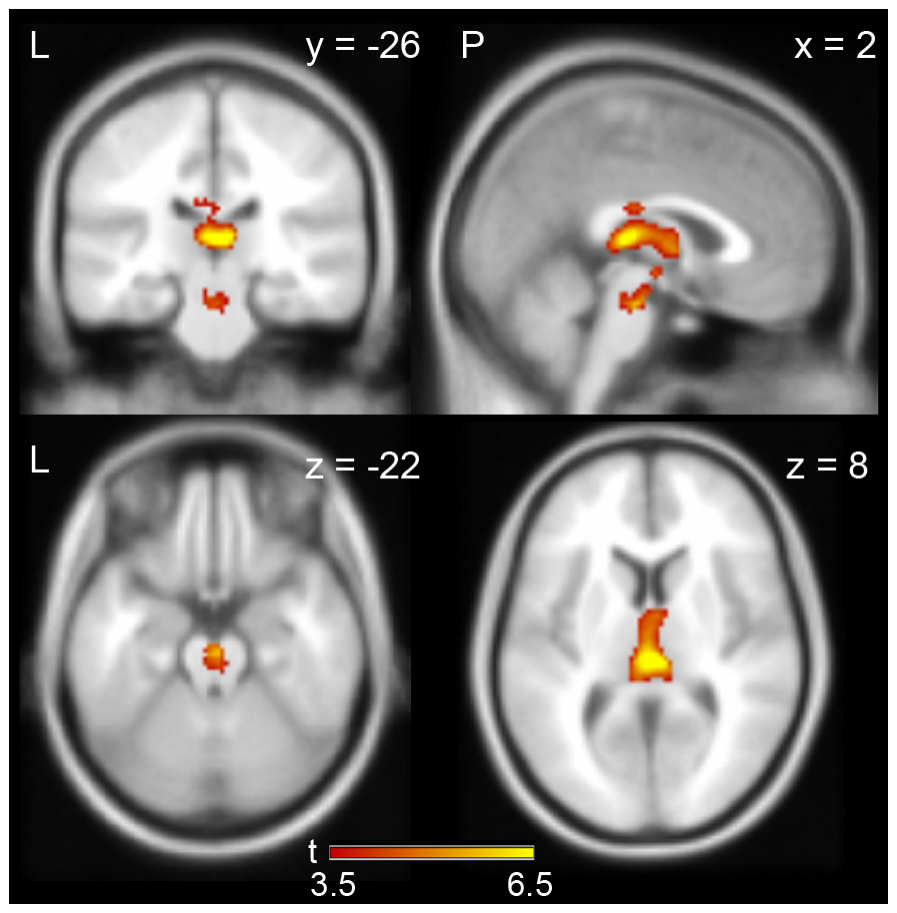
Comparison between the brain regions positively correlated with the slow and fast fluctuation components. Statistical results are superimposed on an averaged MRI (Uncorrected p<0.001, a cluster level FWE of 0.05). The yellow-red color on the brain images indicates the significant difference between the slow and fast fluctuation components (slow > fast). The color bars at the bottom of the figure show t-values between 3.5 and 6.5. The number in the upper right of each slice indicates a MNI coordinate. The letters in the figure indicate the direction of each brain image (L: left; R: right; P: posterior).

A comparison between the brain regions positively correlated with the slow and fast fluctuation components (slow > fast) also revealed a significant difference between components in the middle part of the thalamus and the brain stem (Figure 6 and Table 4, uncorrected p<0.001, extent >135 voxels). Conversely, there was no significant difference in the comparison between the fast and slow fluctuation components (fast > slow).

## Discussion

We conducted simultaneous EEG/fMRI recordings to examine the dynamic relationship between alpha power of EEG and brain activity. We found that the slow and fast fluctuation components of the APTS were correlated with different brain regions in the thalamus, anterior cingulate cortex, and brain stem. These data generally agrees with our hypothesis that the APTS contains mixed dynamics of the alpha power, and suggests that different brain systems may regulate alpha rhythm in parallel.

### Brain regions associated with IMFs of the alpha EEG

We applied the EMD to the APTS to separate it into five IMF components (Figures 1 and 2). In the fMRI analysis, the brain regions positively correlated with each IMF must be categorized into two types of relevant brain regions (see Figure 4 and Tables 2 and 3). The first type mainly consisted of the anterior cingulate cortex and the anterior and lateral part of the thalamus (the results of the IMF1 and 2). The second type predominantly involved the medial and dorsal part of the thalamus and the brain stem, (the results of the IMF3, 4 and 5). The negative correlation between the activity and the IMFs was also categorized into two types. The results of the IMF1 and 2 showed almost no significant brain regions, while that of the IMF3, 4, and 5 showed significant brain regions spreading over the occipital and parietal cortex (see Figure 4). Taken together, these data suggest that the IMF1 and 2 of the APTS are qualitatively different from the IMF3, 4, and 5. There were noticeable differences between the two IMF groups with regard to the thalamus, the anterior cingulate, and the brain stem, supporting the hypothesis that different brain systems may be regulating alpha rhythm in parallel.

Interestingly, the IMF3, 4, and 5 showed no correlation with the activity in the ventral anterior cingulate (vACC), whereas the slow fluctuation of the APTS that corresponded to the IMF3, 4, and 5 explained the brain activity in the vACC (Figures 4 and 5). Thus, the activity in the vACC must include the broad frequency component extending in the range 0–0.04 Hz.

### Brain regions associated with slow and fast fluctuations of the alpha EEG

Our results suggest that the correlation between brain activity and the IMFs must be categorized into two types. The brain regions that correlated with the IMF1 and 2 were noticeably different from that of the IMF3, 4, and 5. Furthermore, the profiles of the power spectrums of the second and the third components had an obvious crossover at 0.04 Hz (see Figure 2B and Figure 4). Therefore, we separated the APTS into slow and fast fluctuation components using a low and high pass filter at 0.04 Hz. Fast fluctuation corresponded to instantaneous increases and decreases in alpha power oscillation, while slow fluctuation corresponded to slower changes depending upon the prominence of alpha oscillation. In the fMRI analysis, the brain regions that correlated with slow and fast fluctuations differed from each other (Figure 5). There were noticeable differences among the thalamus, anterior cingulate cortex, and brain stem, supporting the notion that the brain regions involved in alpha rhythm generation and those indirectly affecting alpha rhythm might coexist and modulate alpha oscillation independently.

Salek-Haddadi et al. [22] stated that “alpha oscillations may be related to three different types of areas: (1) the generators of the cortical rhythm, such as the occipital cortex; (2) areas forming part of the circuit but not directly generating the scalp-detectable rhythms (e.g. thalamus); and (3) other areas correlated with alpha but not causally linked, for example as linked to changes in arousal only.” Brain regions with activity that is positively correlated with fast fluctuation of the alpha rhythm may be located in the lateral part of the thalamus, which is thought to form the thalamocortical circuit that generates the alpha rhythm [43]. These regions may correspond to the second mechanism proposed by Salek-Haddadi et al. [22]. The brain regions that are positively correlated with slow fluctuation may indirectly affect the generation of alpha oscillations through slow changes in brain states, corresponding to the above-mentioned third mechanism.

The arousal level plays an important role in the emergence of the alpha rhythm. Traditionally, the existence of alpha and beta oscillations on the EEG has indicated a wakeful state (e.g [ 24]). Therefore, the positive correlation between brain activity and the slow fluctuation of the APTS may reflect the arousal level. In fact, the brain stem and medial part of the thalamus, the activity of which were correlated with slow fluctuation, form part of the reticular formation that is associated with the arousal level [26]. In the present study, we evaluated the arousal level of the subjects during the experiment using a traditional method [24] (data not shown) and found that the experimental period comprised both awake and drowsy states. Regarding cortical activity during the drowsy state, Horovitz et al. [44] showed increased BOLD fluctuations in the visual cortex during light sleep. We found a negative correlation between the slow fluctuation of the APTS and the occipital-parietal cortex, as the alpha power decreases during the drowsy state. These evidences suggest that the decrease of alpha power during drowsy state may reflect the increase of BOLD signal fluctuations.

In addition to the arousal level, we considered another possibility for the involvement of monoaminergic neurons in the brain stem. The efferent nerves of the monoaminergic systems convey impulses from the brain stem to broad areas of the cerebral cortex [2], [26], [45], [46]. The cortical state changed by monoamine neurotransmitters can occasionally be maintained for seconds to minutes (e.g. [47]–[51]). Considering the time scale of the slow fluctuation component of the alpha power, in which the frequency is below 0.04 Hz (i.e., a period of time longer than 25 seconds), we suggest that this component may also reflect the activity of the diffuse modulator system in the brain stem.

Most monoamine neurons project from the brain stem structures to diffuse brain areas: dopamine from the ventral tegmental area and substantia nigra of the midbrain; serotonin from the raphe nuclei extending throughout the medulla, pons, and midbrain; and noradrenaline from the locus coeruleus in the rostral pons [52]. Although the relevant activation cluster in the brain stem was mainly located in the ventral part of the midbrain and the rostral pons, it is difficult to infer which of the monoamine transmitters might be responsible as the monoamine neurons have reciprocal interactions. However, the cluster explored in the present study is likely to cover these structures.

The positive correlation between the activity in the brain stem and the slow fluctuation, but not the fast fluctuation, suggests that the slow fluctuation may be useful as an index of brain activity in the brain stem. For instance, activity in certain areas of the brain stem, such as the raphe nucleus, is correlated with symptoms of depression [53]. Thus, scalp EEG signals may be useful as biomarkers for such psychiatric symptoms through indirect monitoring of brain stem activity, including that in the raphe nucleus.

The regions of the thalamus that are correlated with slow fluctuation are thought to include the nuclei situated in the dorsomedial part. These nuclei are likely considered to be part of a nonspecific projection system and have a functional role in modulating the degree of activity in the cerebral neocortex [54]. By contrast, regions that are correlated with fast fluctuation are situated more laterally (Figure 5) and are likely to include nuclei with specific projections to the cerebral neocortex and form a thalamocortical loop involved in the generation of alpha oscillations [26], [43], [54]. Furthermore, Schreckenberger et al. [55] reported that the activity of the lateral part of the thalamus was tightly coupled with the alpha power under lorazepam treatment in a PET/EEG study. In that study the correlation of the alpha rhythm to thalamic activity was suggested to reflect thalamic generation of cortical alpha power by the changing of firing patterns in the lateral thalamic nuclei. The coordinates of the lateral thalamus nuclei seem close to the regions that correlated with the fast fluctuation in the present study.

In the present study, the particular regions that were correlated with the fast fluctuation components in contrast to the slow fluctuation components were the superior frontal cortex, and the cerebellum (Figure 5). We believe that the cortical regions in the frontal cortex might be involved in the thalamocortical circuit because of their direct connection with the thalamus [43], [56], [57]. A conclusion is more difficult to reach in terms of the cerebellum, although it is possible that the correlation between the fast fluctuation and the activity in the cerebellum might reflect activity of cerebrocerebellar interaction, as the cerebral cortex and cerebellum have a crossed connection and the regions in the cerebral cortex and cerebellum illustrated in the present study were lateralized to the right and left, respectively. Of course, these interpretations should be explicitly tested in the future.

The cingulate cortex has a direct connection with various thalamic nuclei [58]–[60]. Although it was difficult to precisely identify the thalamic nuclei in detail using the low spatial resolution of the present study, we believe that the differential involvement of the fast and slow components in the cingulate cortex might reflect differences in thalamic connections. Furthermore, the slow and fast fluctuation components were associated with the brain activity in the dorsal and ventral part of anterior cingulate cortex (dACC/vACC), respectively (Figure 5). The dACC is considered to be involved in cognitive processes, while the vACC in emotional regulation [61], [62]. This implies that the fast fluctuation of the APTS may be associated with cognitive processes, and the slow fluctuation may be relevant to emotional processes.

### Comparison with previous simultaneous recording experiments

The results in Figure 3 are mostly consistent with those of previous studies [7], [10]–[12], [15]–[17]. Although some studies reported no correlation between BOLD signals in the thalamus and alpha oscillations [13], [63] and a negative correlation between the glucose metabolic rates in the thalamus and averaged alpha power [8], [9], recent studies have generally shown positive correlations between alpha power fluctuation and BOLD signals in the thalamus and negative correlations in the occipitoparietal cortex.

### Characteristics of APTS fluctuations

In the present study, we tried to characterize two different aspects of alpha power fluctuation, that is, the fast fluctuation corresponding to instantaneous increases and decreases in alpha power oscillation, such as waxing and waning [23], and the slow fluctuation corresponding to slower changes depending upon the ease of alpha oscillation. The cutoff frequency between the fast and slow components was determined based on the brain patterns associated with IMFs using the EMD (see Figures 2 and 4). However, since the occupied frequency of each IMF varied across the subjects, the border of the slow and fast components must be considered as a rough indication.

Brain activity in a resting state, with eyes closed or while looking at a fixed point, was recently examined by looking at changes in BOLD signals (cf. [64]). The majority of the studies postulate that BOLD signal fluctuation of the default mode network, including the posterior cingulate cortex and the medial frontal cortex, is in a frequency range of less than 0.1 Hz [64]–[68]. Niazy et al. [34] reported that resting-state networks are not merely described by slow spontaneous fluctuations (∼0.015 Hz), but by broadband processes that indicate temporal coherences across a frequency spectrum, especially in the range of 0.02–0.05 Hz. Interestingly, the frequency range in the present study (<0.04 Hz) is included in that of the default mode network.

In terms of the relationship between spontaneous fluctuation of BOLD signals and EEGs, using a concurrent EEG and fMRI with group independent component analysis, Bridwell et al. [69] reported positive associations with alpha rhythm within the thalamus and medial frontal gyrus, and negative associations between frontal, parietal, temporal, and limbic fMRI regions, and EEG alpha. Furthermore, an MEG study demonstrated that the default mode network was identified using alpha band data [70]. In addition, He et al. [71] reported that the slow cortical potentials measured by electrocorticography in humans show a correlation structure similar to that of the resting state network in BOLD fluctuations. These findings suggest that the brain network affecting alpha rhythm generation and the resting state may share a common fluctuation mechanism.

Although we have discussed the physiological aspects of the APTS and spontaneous BOLD fluctuation, it is unlikely that alpha power fluctuation solely reflects spontaneous fluctuation. In general, the alpha rhythm is changed by spontaneous fluctuation, and both the internal state of subjects and external stimuli. Alpha oscillation was reportedly increased by sounds containing inaudible high-frequency components associated with activation of the deep-lying brain regions, and both were significantly correlated [10]. Therefore, the slow fluctuation of alpha oscillation may be useful as a convenient objective marker to monitor the deep-lying brain structures, including the brain stem and medial thalamus.

## Conclusions

We showed that the dynamics of the alpha power were positively correlated with brain activity in the deep-lying brain regions, the thalamus and brain stem. Moreover, we showed that the slow and fast fluctuation components of the transient alpha power were correlated with particular brain regions (the slow component with the medial part of the thalamus and the brain stem, and the anterior cingulate cortex; the fast component with the lateral part of the thalamus and the anterior and middle cingulate cortex). These results support our hypothesis that the APTS consist of different dynamics of modulation of alpha oscillation, and that different subcortical structures contribute to slow and fast modulations of alpha spectra.
